# Crashworthiness Performance and Multi-Objective Optimization of Bi-Directional Corrugated Tubes under Quasi-Static Axial Crushing

**DOI:** 10.3390/ma17163958

**Published:** 2024-08-09

**Authors:** Liuxiao Zou, Xin Wang, Ruojun Wang, Xin Huang, Menglei Li, Shuai Li, Zengyan Jiang, Weilong Yin

**Affiliations:** 1 Center for Composite Materials and Structures, Harbin Institute of Technology, Harbin 150080, China; zlx19851196807@163.com (L.Z.);; 2 Department of Engineering Mechanics, Kunming University of Science and Technology, Kunming 650031, China; 3 Aerodynamics Research Institute, AVIC (Aviation Industry Corporation of China), Harbin 150080, China; 4 National Key Laboratory of Science and Technology on Advanced Composites in Special Environments, Harbin Institute of Technology, Harbin 150080, China

**Keywords:** bi-directional corrugated tubes, axial crushing, energy absorption, stable plateau force, multi-objective optimization

## Abstract

Longitudinal corrugated tubes (LCTs) exhibit stable platform force under axial compression but have low specific energy absorption. Conversely, circumferential corrugated tubes (CCTs) offer higher specific energy absorption but with unstable platform force. To overcome these limitations, this paper introduces a novel bi-directional corrugated tube (BCT) that amalgamates the strengths of both the CCT and LCT while mitigating their weaknesses. The BCT is formed by rolling a bi-directional corrugated structure into a circular tubular form. Numerical simulations of the BCT closely align with experimental results. The study further examines the influence of discrete parameters on the BCT’s performance through simulations and identifies the tube’s optimal design using the integral entropy TOPSIS method. A full factorial experimental approach is then employed to investigate the impact of radial amplitude, axial amplitude, and neutral surface diameter on the crushing behavior of the BCT, comparing it with the CCT and LCT. The results reveal that increasing $A_{i}$ enhances the axial resistance of the structure, while increasing $A_{j}$ reduces the buckling effect, resulting in a higher specific energy absorption and lower ultimate load capacity for the BCT compared to the CCT and LCT. A simultaneous multi-objective optimization of the CCT, LCT, and BCT confirms that the BCT offers superior specific energy absorption and ultimate load capacity. The optimal configuration parameters for the BCT have been determined, providing significant insights for practical applications in crashworthiness engineering.

## 1. Introduction

In the quest for light weight and high energy absorption structures, thin-walled tubes have emerged as the predominant choice for energy absorption applications [1,2,3], notably in spacecraft landers [4], medical apparatus [5], robots [6], and automobiles [7,8,9]. An exemplary energy-absorbing structure must possess not only a superior capacity to absorb energy but also maintain a consistent and stable reaction force throughout its deformation process. This combination of attributes ensures effective energy dissipation while minimizing the impact on the system or its occupants.

Thin-walled tubes subjected to axial compression are frequently utilized as crash boxes behind car bumpers, owing to their extended deformation stroke and superior energy absorption. The performance of these tubes is predominantly influenced by the topological characteristics of their cross-sections and their subsequent evolution, which can be meticulously controlled through mathematical functions or curves. This approach allows for the facile generation of diverse cross-sectional profiles by adjusting specific parameters of the control function or curve. While Wu et al. [10] introduced the use of the Fourier function in cross-section design, it was noted that Fourier cross-section tubes may have limited applicability in sandwich structures due to non-coincidence of the tangent points of their concave sections along the same circumference. Alternatively, Deng et al. [11,12] proposed circumferentially corrugated tubes, which exhibit enhanced energy absorption capabilities due to the increased structural resistance conferred by their complex cross-sections. Similarly, Li et al. [13] and Eyvazian et al. [14] introduced transversely corrugated square tubes with sinusoidal profiles, demonstrating higher specific energy absorption compared to conventional square tubes. However, it is noteworthy that these circumferential corrugated tubes, despite their high energy absorption, often produce force–displacement curves characterized by high initial peak loads and unstable plateau forces. This instability stems from the repeated buckling and bending failures of axially loaded tubes.

Extensive exploratory studies have been conducted to mitigate the initial peak force and enhance the stability of the crushing process in thin-walled structures. Singace et al. [15] experimentally investigated the energy absorption characteristics of corrugated tubes, introducing corrugations to enforce plastic deformation and improve the uniformity of the load–displacement behavior under axial loading. Eyvazian et al. [16] explored the impact of corrugation on the crushing behavior of circular aluminum tubes, focusing on energy absorption and damage patterns. Their findings indicated that longitudinal corrugated tubes promote more predictable and controllable collapse patterns. The controllability and predictability of the collapse patterns are crucial for applications where precise energy management is essential, such as in automotive safety systems, aerospace structures, and protective packaging. By utilizing corrugated tubes, engineers can design systems that more effectively dissipate impact energy, reducing the risk of injury or damage to occupants or surrounding structures. Consequently, these tubes are considered promising candidates for controlled energy absorption devices and have garnered significant research interest.

Dong et al. [17] designed flexure-inducing tubes based on the modal analysis of straight-walled triangular tubes, enabling folding according to specific modes by strategically presetting convex and concave structural forms. Chen et al. [18] analyzed the non-linear elastic–plastic behavior of circular tubes with corrugated profiles using finite element methods, providing insights into their deformation mechanisms and energy absorption characteristics. Eyvazian et al. [19] conducted both experimental and theoretical examinations of corrugated tubes under axial loading, validating their findings and reinforcing the understanding of their crushing behavior. Alkhatib et al. [20] focused on the additive manufacturing and experimental testing of sinusoidal corrugated tubes under quasi-static loading conditions. Imai et al. [21] employed isogeometric analysis and experiments to study the effect of contour curvature on the energy absorption characteristics of cylindrical corrugated tubes. Wu et al. [22] proposed sinusoidal corrugated tubes designed to control collapse modes and minimize the initial peak crushing force and load fluctuation. Their findings revealed that while these tubes exhibited more uniform force–displacement curves, they significantly reduced energy absorption compared to conventional straight circular tubes. Inspired by the concept of functionally graded materials, Yao et al. [23] developed novel hybrid corrugated tubes with an amplitude profile that decreases progressively from large to small during compression. This design was found to enhance energy absorption capacity compared to ordinary corrugated tubes. Finally, Ha et al. [24] introduced an innovative tubular corrugated structure, modeled after the profile of a coconut tree. This design aimed to improve energy absorption, reduce the initial peak crushing force, and stabilize the crushing process.

Despite their remarkable ability to effectively mitigate initial peak forces and plateau force fluctuations, corrugated tubes exhibit a trade-off in the form of reduced specific energy absorption. The results indicate that deformation behavior can be stabilized and load–displacement curves, as well as energy absorption, can be controlled by introducing corrugations with varying distances. In summary, these corrugated tubes demonstrate significant potential for reducing initial peak forces and enhancing deformation stability, which has garnered increasing attention. However, it is important to note that the specific energy absorption of these corrugated tubes is diminished.

**Figure 1:**
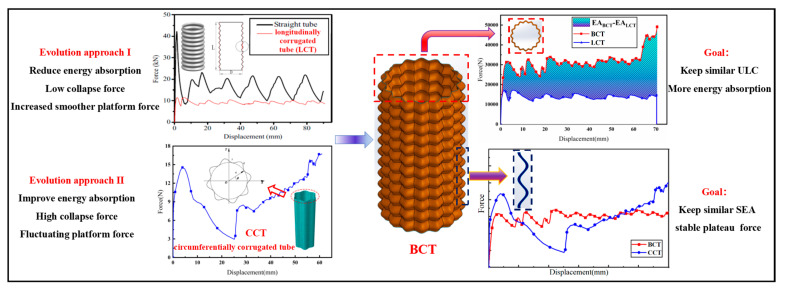
Comparing the force–displacement curves of a CCT and an LCT under axial compressive loads, and in order to keep similar specific energy absorption and obtain a more stable plateau force, a BCT structure is proposed.

As illustrated in Figure 1, longitudinally corrugated tubes (LCTs) exhibit a low collapsing force, smooth plateau force, and reduced energy absorption under axial compression. In contrast, circumferentially corrugated tubes (CCTs) demonstrate higher energy absorption, accompanied by a higher collapsing force, and a fluctuating plateau force. Based on the shape of the force–displacement curve under quasi-static compression, energy absorbers can be broadly classified into two categories: those with a nearly “flat-topped” load–displacement curve and those with an initial peak force followed by a steeply decreasing curve. Obviously, the force–displacement curve of the LCT aligns with the former type, characterized by a nearly “flat-topped” profile, whereas the CCT exhibits the latter type, marked by an initial peak force followed by a steep decline. This dichotomy underscores the contrasting force–displacement characteristics of the LCTs and CCTs. An analysis of the cross-sectional shapes reveals that the ripples in the LCTs are present on the sidewall, while those in the CCTs appear on the cross-section. By integrating sidewall corrugations and cross-section corrugations, a bi-directional corrugated tube (BCT) can be developed. Notably, BCTs exhibit a unique tunability, as their properties can be modulated by adjusting the amplitudes of the cross-sectional and sidewall corrugations. When the cross-sectional corrugation amplitude of the BCT is zero, it degenerates into an LCT; similarly, when the sidewall corrugation amplitude of the BCT is 0, it degenerates into a CCT. This adaptability suggests the feasible of identifying BCT structures with relatively stable plateau forces and higher energy absorption.

In this study, BCTs were fabricated using 3D printing and subjected to axial compression tests to investigate their deformation mechanisms. To further explore the deformation and energy-absorbing properties of BCTs under axial compression, a comprehensive finite element model was developed using the commercial software ABAQUS. Initially, discrete parameters were parameterized based on the finite element method to assess the crashworthiness of these BCTs. The integral entropy TOPSIS method was then employed to evaluate and select the optimal discrete parameters for crashworthiness. Subsequently, the full factorial design method was utilized to examine the effects of continuous parameters such as circumferential ripple amplitude $A_{i}$, axial ripple amplitude $A_{j}$, and neutral plane diameter D. The results were compared with those of CCTs and LCTs to underscore the advantages of BCTs. Finally, multi-objective optimization was performed for CCTs, LCTs, and BCTs to highlight the combination of high specific energy absorption and stable plateau force in BCTs, ultimately determining the optimal geometric parameters for the tubes.

## 2. Materials and Methods

### 2.1. Geometric Features

**Figure 2:**
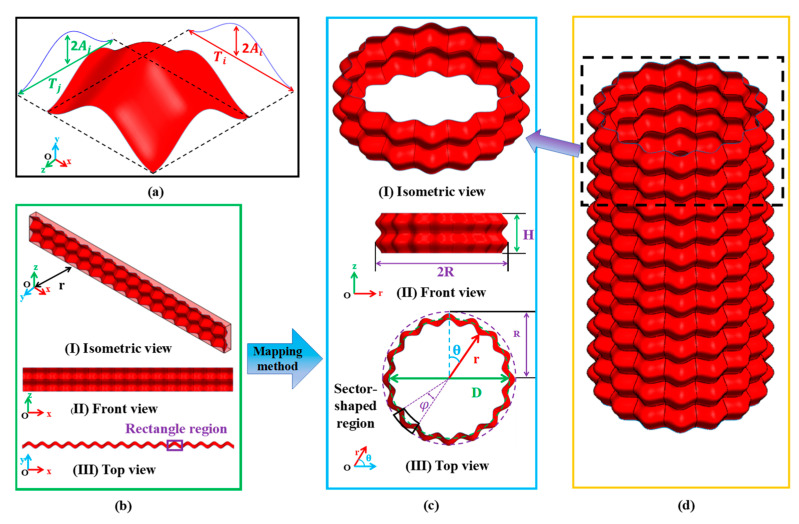
Geometry modeling process for the BCT: (**a**) bi-directional corrugated structure, (**b**) structure with 12 × 2 cells, (**c**) BCT modeling by the mapping method, (**d**) geometric modeling of the BCT.

Figure 2a meticulously illustrates the bi-directional corrugated structure, showcasing its unique undulating patterns in two perpendicular directions. Within this context, the Cartesian coordinate system, defined by the axes $\left(x,y,z\right)$, serves as a fundamental reference framework for elucidating the geometry of the structure. The governing equations in the x and z directions are:

(1) $$y_{i}=A_{i}\times cos\left[2\pi Nx/\left(\pi D\right)\right]$$

(2) $$y_{j}=A_{j}\times cos\left[2\pi Mz/H\right]$$

The amplitudes of the ripple curves in the *x*-axis and *z*-axis directions are denoted as $A_{i}$ and $A_{j}$, respectively. N represents the number of units in a single-layer BCT, while D denotes the diameter of the neutral plane of the BCT. M signifies the number of layers in the BCT, and H indicates the height of the BCT.

To fully leverage the superior mechanical properties of bi-directional corrugated structures within tubular frameworks, the bi-directional corrugated structure is integrated as the unit element within the tubular structure to enhance its resistance to axial compression. The fabrication process of the BCT is illustrated in Figure 2. It is evident that the 3D structure depicted in Figure 2b (I) is transformed into a circular tubular form as shown in Figure 2c (II). The complete BCT consists of N and M bi-directional corrugated units arranged in the circumferential and axial directions, respectively. Here, N and M denote the number of bi-directional corrugated structural units aligned in the x and z directions within the Cartesian coordinate system. Figure 2b presents the isometric, front, and top views of this structure, with N and M values of 16 and 2, respectively. The distance from the XOZ plane is denoted as r, representing the inner diameter of the BCT, as illustrated in Figure 2b (I). The bi-directional corrugated structure in Figure 2b is bent to form the circular tubular BCT depicted in Figure 2c. The coordinates of the BCT in the column coordinate system are represented as $\left(r,\theta \right)$. The radial thickness of the BCT, shown in Figure 2c (II), is determined by $2(A_{i}+A_{j})$, which also represents the thickness of the bi-directional corrugated structural unit along the x-axis. Furthermore, the bending angle φ is calculated as *φ* = 2*π*/*N*. The geometric model of a twelve-layer BCT is illustrated in Figure 2d, with specific geometric parameters detailed in Figure 2c. For clarity, the wall thickness (t) is 1 mm, and the length (H) is 120 mm.

### 2.2. Crashworthiness Indicators

This section examines six key crashworthiness criteria to offer a comprehensive understanding of energy absorbers through various metrics. These metrics are defined as follows.

1. Energy absorption (EA): This is defined as the area under the force–displacement curve, given by:
  (3) $$EA=\int_{0}^{s}F\left(x\right)dx$$
  where F(s) denotes the loading force as a function of displacements. S represents the effective stroke, which is numerically defined as 70% of the tube length.
2. Specific energy absorption (SEA): This metric represents the energy absorption per unit mass and indicates the energy absorption efficiency of the structure. It can be expressed as:
  (4) $$SEA=\frac{EA}{m}=\frac{\int_{0}^{S}F\left(x\right)dx}{m}$$
3. Initial peak crushing force (IPCF): This refers to the maximum force attained on the force–displacement curve following the elastic phase. The force at this peak is termed the IPCF.
4. Mean crushing force (MCF): MCF is defined as the ratio of EA to the effective stroke S. It can be quantified by the following equation:
  (5) $$MCF=\frac{EA}{S}=\frac{\int_{0}^{S}F\left(x\right)dx}{S}$$
5. Crushing force efficiency (CFE): ratio of MCF to $F_{max}$.
  (6) $$CFE=\frac{MCF}{F_{max}}$$
6. Undulation of load carrying capacity (ULC): ULC quantifies the smoothness of the force–displacement curve by calculating the ratio of the work done due to deviations of the actual load from the MCF to the EA. A smaller ULC value signifies a more uniform and smoother energy absorption process.
  (7) $$ULC=\frac{\int_{0}^{S}\left|F\left(x\right)-MCF\right|dx}{\int_{0}^{S}F\left(x\right)dx}$$

### 2.3. Compression Test

**Figure 3:**
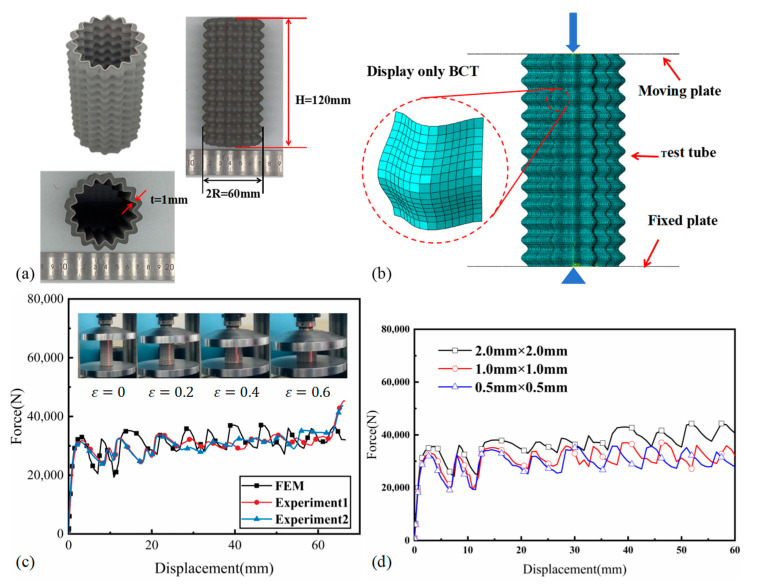
(**a**) Schematic diagram of the BCT specimen. (**b**) FE model of the tube under axial impact loading. (**c**) Force–displacement curves of experimental and numerical results of the BCT. (**d**) Mesh-size validation for the force–displacement of the BCT under axial compression.

The material used for the BCT was stainless steel 316 L, with the following mechanical properties: [25] density $\rho =7980\;kg/m^{3}$, Young’s modulus E=171 GPa, yield stress $\sigma_{y}=552\;MPa$, and Poisson’s ratio ν=0.3. Given that quasi-static loading conditions are simulated, strain rate effects are not considered into the finite element modeling. To evaluate the stability of the BCT platform under axial compression and its capacity for energy absorption, the BCT specimen was prepared as illustrated in Figure 3a. The specimen, fabricated using additive manufacturing techniques, features a height of 120 mm and a wall thickness of 1 mm. The chosen parameters for the number of corrugations N and the number of layers M were 16 and 12, respectively. Additionally, both $A_{i}$ and $A_{j}$ were set to 2 mm, and the diameter of the neutral plane (D) was specified as 51 mm.

The degradation history of the BCT, as depicted in Figure 3c, provides valuable insights into its behavior under axial compression loads applied at a quasi-static rate of 5 mm/min. As compression displacement increases, the BCT undergoes a controlled deformation process, with layers progressively collapsing from the base upwards. This compression includes the formation of lobes, with 12 folds present and observable. The force–displacement curves for the BCT, as shown in Figure 3c, demonstrate a high degree of correlation between experimental and simulated data. These curves reveal a stable plateau force throughout the compression process. Notably, the experimental force–displacement curves exhibit reduced fluctuation, with the number of oscillations corresponding to the number of pre-folded layers. This finding further supports the notion that the BCT’s deformation is well ordered and controlled, leading to a more stable and predictable performance under compression.

### 2.4. FE Modeling

The crashworthiness of the BCT was assessed using the finite element analysis software ABAQUS/Explicit. The finite element model, as depicted in Figure 3b, consists of three components: the moving plate, the fixed plate, and the test tube. Initially, all degrees of freedom of the rigid plate are coupled to a reference point, where boundary conditions are applied. The upper rigid plate is subjected to a vertical downward displacement with a velocity of 1 m/s [22], while the lower rigid plate is fixed in all six degrees of freedom. Meshing is performed using 4-node simplified integral linear quadrilateral elements (S4R). The numerical model incorporates generalized contact with a friction coefficient of 0.3 to accurately represent the interaction between the rigid plate and the tube. To balance computational efficiency with accuracy, a cell size of 1.0 mm×1.0 mm is utilized. For the rigid plates, which are modeled as discrete rigid shells, no cell properties are assigned, and the mesh size for these shells is set to 4 mm. This approach allows for a detailed analysis of the BCT’s deformation and energy absorption mechanisms while keeping the overall simulation time manageable.

As shown in Figure 3c, the force–displacement curves obtained from the FEM exhibit an elastic phase similar to the experiment. After reaching a similar IPCF, the curve fluctuates up and down in the plastic plateau segment in small magnitudes. The overall agreement between the FEM and experimental force–displacement curves, particularly in the elastic and plastic plateau segments, provides confidence in the validity and accuracy of the simulation results. These results can then be used to further analyze the crashworthiness of the BCT material.

Figure 3c illustrates a comparison between the experimental force–displacement data and numerical results, demonstrating a strong concordance between the finite element simulations and the experimental observations. The response curves plotted in Figure 3c exhibit comparable trends and fluctuation characteristics. The IPCF for specimen 1 and specimen 2 are 31.76 kN and 31.26 kN, respectively, while the simulated IPCF is 32.95 kN, as detailed in Table 1. This close correspondence further reinforces the validity of the finite element model. Moreover, the discrepancies in EA and MCF between experimental and simulated data are within 3%, highlighting the precision of the simulation in capturing the essential characteristics of the BCT. These findings provide strong evidence that the finite element model was successfully calibrated to accurately represent the mechanical behavior of the BCT under the given loading conditions.

## 3. Discussion

### 3.1. Discrete Parametric Analysis

#### 3.1.1. Effect of N and M

**Figure 4:**
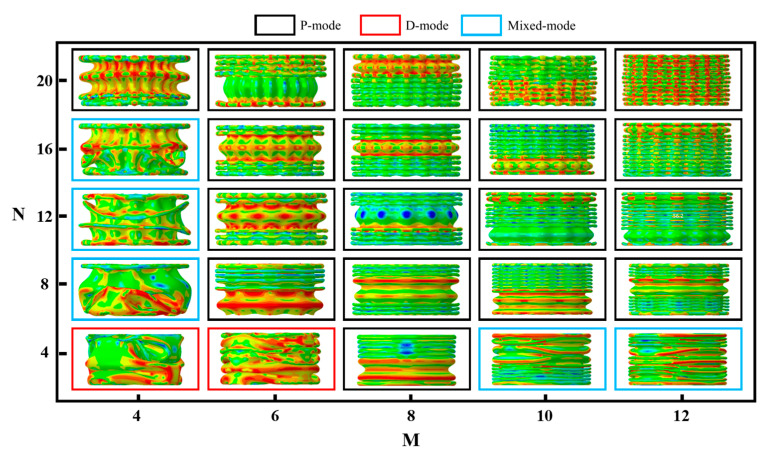
Distribution of deformation modes.

Figure 4 provides a detailed analysis of 25 design cases with varying values for *N* and *M*, revealing that the deformation modes of the BCTs predominantly fall into three distinct categories: progressive flexure mode (P-mode) [26], diamond mode (D-mode), and mixed mode (*M*-mode). When N=20, the collapse consistently occurs in P-mode, regardless of the value of M. This observation suggests that a higher N value promotes a more uniform progressive flexure due to increased rigidity and stability provided by the higher number of cells along the tube’s length. For N=4, both M4 and M6 exhibit D-mode deformation, similar to conventional corrugated tubes (CTs). The fewer layers in these configurations result in larger individual cell heights, making the structure more prone to diamond-shaped deformations under compression. As the number of layers increases (e.g., M8), the deformation transitions to P-mode. This indicates that a higher layer count, resulting in smaller cell heights, enhances the structure’s ability to undergo progressive flexure rather than localized diamond-shaped buckling. Interestingly, in configurations with a greater number of layers (e.g., M10 and M12), a mixed deformation mode is observed. Initially, the structure undergoes P-mode deformation, followed by a transition to D-mode. This behavior can be attributed to the balance between the increased structural integrity provided by the additional layers and the inherent tendency of the structure to buckle in a diamond pattern under certain conditions. For intermediate values (8≤N≤16), the deformation mode shifts from mixed mode to P-mode as N increases. This shift indicates a critical threshold, where the structural configuration favors progressive flexure over mixed deformation, likely due to the optimized balance between cell height and layer count. These observations highlight the complex interplay between the geometric parameters N and M in determining the deformation behavior of BCTs. By leveraging this understanding, engineers and designers can tailor the geometric configuration of BCTs to achieve desired mechanical properties for specific applications.

**Figure 5:**
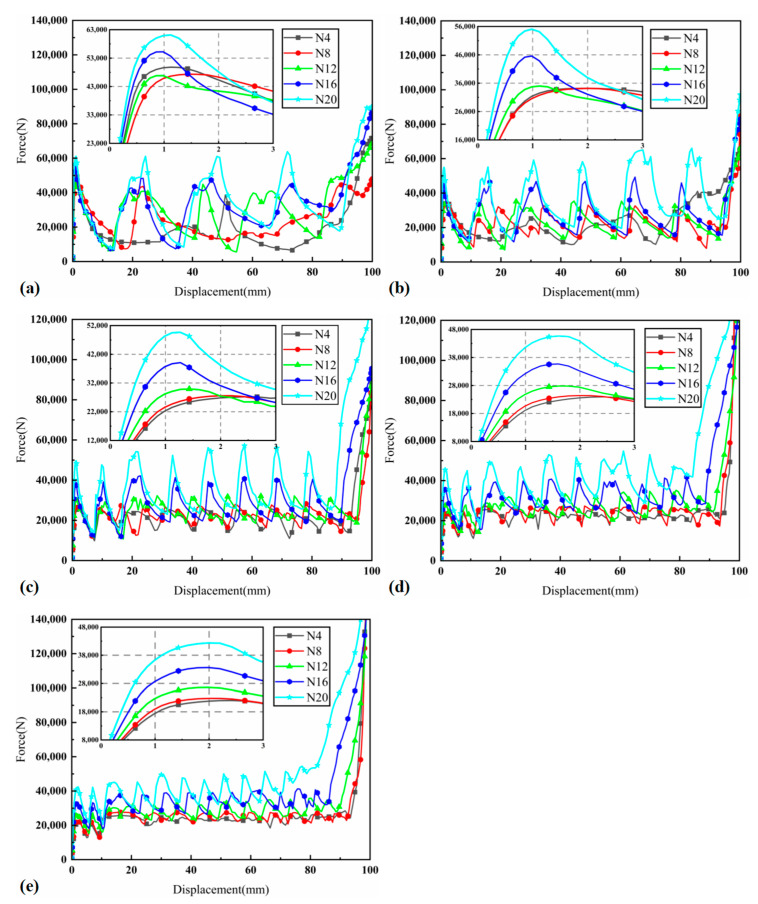
Load–displacement diagram of BCTs: (**a**) M4, (**b**) M6, (**c**) M8, (**d**) M10, and (**e**) M12.

Figure 5 presents the load–displacement curves for 25 different cases, providing insight into the mechanical performance of BCTs under varying geometric parameters. Notably, the initial stiffness of the BCTs is observed to increase with a larger N. This phenomenon can be attributed to the greater number of cells along the tube’s length, which enhances the overall structural rigidity and resistance to initial deformation. Conversely, the initial peak crushing force (IPCF) decreases with an increase in M or a decrease in N. This indicates that BCTs with more layers (higher M) or fewer cells along the length (lower N) are less capable of sustaining high peak forces, likely due to the reduced height of individual cells, which leads to earlier onset of buckling and less force being required for deformation. Except for cases N4M4 and N8M4, the peaks of the BCT curves are closely aligned, reflecting effective energy absorption capabilities. These two exceptions can be explained by their unique configurations where the combination of N and M results in less optimal energy dissipation characteristics, possibly due to localized buckling or irregular deformation patterns. Furthermore, as N decreases, the load–displacement curves exhibit reduced fluctuation, leading to smoother energy absorption. This behavior suggests that BCTs with fewer cells along the length distribute the applied load more evenly across the structure, thereby avoiding abrupt changes in force and resulting in a more stable energy absorption process. Specifically, for lower N values (e.g., N4), the curves show more pronounced peaks and valleys, indicating intermittent energy absorption due to localized buckling events. As N increases (e.g., N12 and above), the curves become smoother, with less pronounced fluctuations, suggesting a more uniform deformation process and continuous energy absorption. These findings underscore the importance of optimizing both N and M to achieve desired mechanical properties, particularly in applications requiring high energy absorption and minimal force fluctuations.

**Figure 6:**
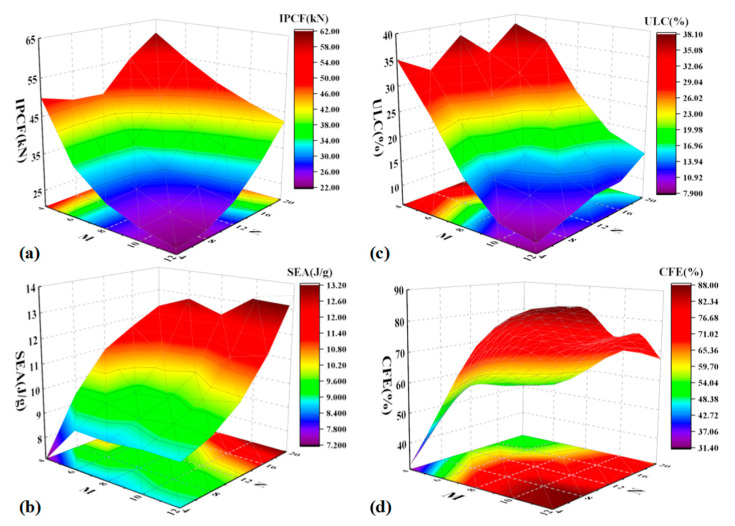
Crashworthiness parameter chart of the BCT: (**a**) IPCF, (**b**) SEA, (**c**) ULC, and (**d**) CFE.

Figure 6 presents a detailed analysis of the crashworthiness parameters of the BCTs, offering insights into their performance under various geometric configurations. IPCF increases with a decrease in M or an increase in N. This indicates that BCTs with fewer layers (lower M) or more cells along the length (higher N) can sustain higher peak forces before deformation. This behavior is likely due to the increased structural integrity provided by a higher number of cells, which enhances resistance to initial impact forces. SEA rises with increasing values of both M and N, though the influence of N is more pronounced. This suggests that while both parameters contribute to the energy absorption capacity of the BCTs, the number of cells along the length (N) plays a more critical role. By fostering a more distributed pattern of energy absorption, a higher N value leads to more distributed energy absorption, enhancing the overall crashworthiness of the structure. ULC shows minimal sensitivity to changes in N but increases significantly with higher M. This indicates that the layer count (M) is a more crucial factor in determining the maximum load-bearing capacity of the BCT before failure. Higher M values enhance the overall structural strength, allowing the BCT to absorb more energy before reaching its ultimate load. CFE generally increases with larger values of M. This reflects the improved ability of the BCT to absorb energy efficiently during deformation. Higher M values result in a more stable and controlled collapse mechanism, enhancing the overall energy absorption efficiency of the structure.

The impact resistance of the BCT is also illustrated in Figure 6. The results indicate that BCTs with higher values of N and M exhibit superior impact resistance, demonstrating their effectiveness in dissipating impact energy and minimizing damage. A higher *N* increases IPCF due to the added structural elements, while a lower M decreases IPCF because of fewer layers to distribute the load.

Both M and N contribute positively to increasing SEA, but the impact of N is more significant, indicating a strong dependence on the longitudinal configuration of the BCT. On the other hand, the insensitivity of ULC to N suggests that the overall lengthwise configuration is less critical, whereas higher M enhances the BCT’s ultimate load capacity significantly. Furthermore, larger M values result in higher CFE, showcasing better energy absorption capabilities and efficient crash management.

#### 3.1.2. Selection Method and Results

Selecting the BCT from the 25 proposed designs presented a challenge due to the multiple evaluation criteria involved. To address this challenge, the Technique for Order of Preference by Similarity to Ideal Solution (TOPSIS) method [27], widely recognized for its efficacy and versatility across various domains, was employed. This method effectively mitigates subjective bias and accommodates both quantitative and qualitative data [27]. Utilizing the integral entropy TOPSIS approach [28], it is possible to derive the weights of the indicators $w_{i}$ and calculate the relative proximity $Q_{j}$. Detailed procedural steps are provided in Appendix A.

Following the exclusion of six BCTs with ULC exceeding 30%, the remaining 19 BCTs were assessed based on crashworthiness metrics. Among these, SEA and CFE were identified as advantageous criteria, with weights assigned as 0.71 and 0.29, respectively. The relative distances between the tubes were calculated and are detailed in Appendix B (Table A1). Notably, M12 consistently yielded the highest $Q_{j}$ irrespective of variations in N. When varying M, N20 and N16 consistently exhibited the two highest $Q_{j}$ values. However, ULC for N20 was disproportionately high compared to N16. Consequently, for all subsequent analyses, N16M12 was selected as the optimal configuration.

### 3.2. Parametric Analysis of Continuous Type Parameters

#### 3.2.1. Deformation Patterns

Table 2 outlines the design framework involving three variables, each evaluated across six levels, culminating in a total of 216 design cases. These cases incorporate various values for $A_{i}$, $A_{j}$, and D, which are subsequently simulated. An interesting aspect of this design framework is the ability to transform the BCT into other common structural forms by setting specific values for $A_{i}$ and $A_{j}$. For instance, when both $A_{i}$ and $A_{j}$ are set to 0, the BCT effectively transforms into a circular tube (CT). This transformation allows for a direct comparison between the performance of the BCT and that of a traditional circular tube. Likewise, setting $A_{i}$ to 0 results in the BCT resembling a LCT, while setting $A_{j}$ to 0 yields a CCT.

**Figure 7:**
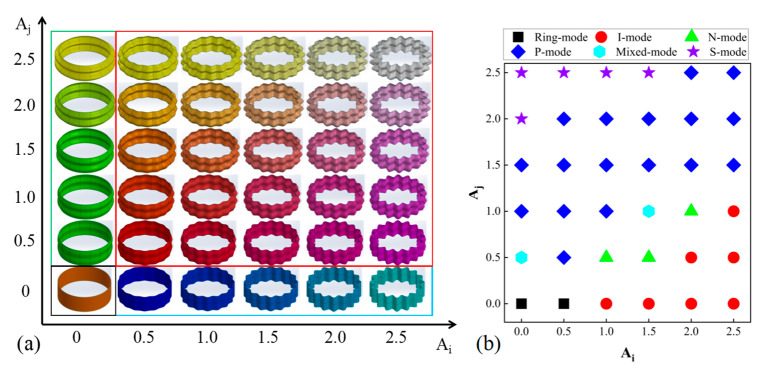
(**a**) BCT conformational distribution (D60) and (**b**) distribution of deformation patterns (D60).

Figure 7 presents a detailed depiction of the conformational distribution of BCTs (D60). Figure 7a displays the configuration of BCTs with 1 CT illustrated in black wireframe, 5 LCTs in green wireframe, 5 CCTs in blue wireframe, and 25 BCTs in red wireframe. Figure 7b elucidates the distribution of deformation modes observed in the D60 configuration. When $A_{i}$ is 0, LCTs exhibit three distinct deformation modes. Similarly, when $A_{j}$ is 0, CCTs exhibit two deformation modes. This analysis confirms that BCTs are capable of exhibiting a total of five distinct deformation modes.

**Figure 8:**
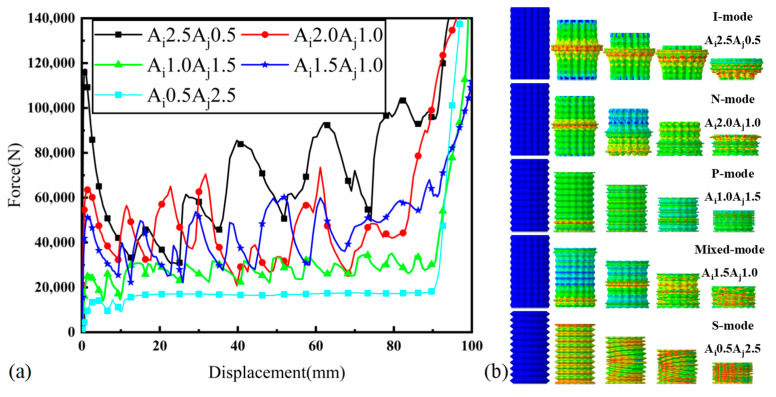
(**a**) Representative force–displacement curves and (**b**) deformation history diagram of representative deformation patterns.

Figure 8 illustrates the deformation modes and corresponding force–displacement curves for the BCT with D60, highlighting distinct behaviors under different configurations. Observed in $A_{i}2.5A_{j}0.5$, this mode is characterized by high IPCF and unstable energy absorption within the plastic deformation region. The force–displacement curves show significant fluctuations, indicating inefficient energy absorption. This behavior is consistent with previous reports [29], where the structural instability leads to rapid changes in force. When $A_{i}2.0A_{j}1.0$, the BCT exhibits *N*-mode, resulting in a reduction in IPCF. However, the force–displacement curves still display pronounced fluctuations. This indicates that while the peak force is lower, the energy absorption process remains unstable, leading to less efficient performance [18]. In the configuration $A_{i}1.0A_{j}1.5$, the BCT exhibits P-mode, characterized by a lower IPCF and reduced fluctuations in the force–displacement curves. The plastic section shows a plateau force marginally exceeding IPCF, indicating a more stable and efficient energy absorption mode. This mode ensures that the energy is absorbed progressively, minimizing the risk of sudden structural failure. Observed in $A_{i}1.5A_{j}1.0$, this mode demonstrates localized diamond deformation following P-mode. The force–displacement curves are similar to N-mode but with reduced fluctuation, indicating a combination of progressive and localized deformation mechanisms. This mixed behavior provides a balance between stability and energy absorption efficiency. When the corrugation parameters are set to $A_{i}0.5A_{j}2.5$, the BCT exhibits S-mode, characterized by concurrent deformation of all units. This results in very smooth force–displacement curves with an ultimate load capacity (ULC) below 10%. The uniform deformation leads to highly efficient energy absorption with minimal fluctuations, making this mode the most stable and effective among the observed configurations. Overall, the analysis shows that by carefully selecting the corrugation parameters $A_{i}$ and $A_{j}$, engineers can fine-tune the deformation modes and energy absorption capabilities of the BCT to suit specific design requirements.

#### 3.2.2. Crashworthiness Analysis

**Figure 9:**
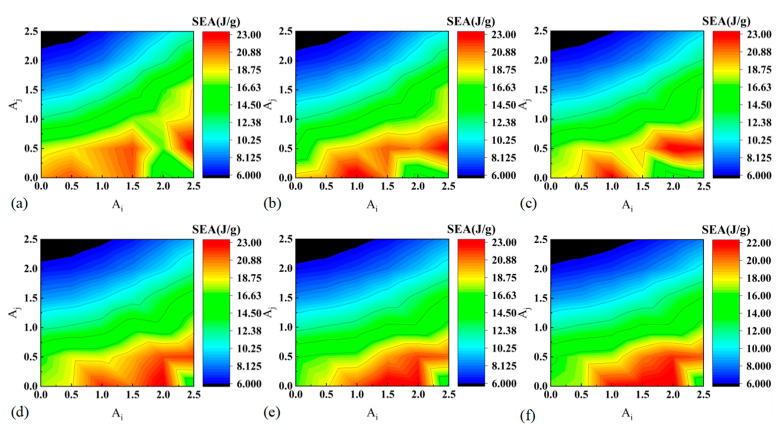
Contour plots of SEA: (**a**) D48, (**b**) D52, (**c**) D56, (**d**) D60, (**e**) D64, and (**f**) D68.

Figure 9 presents the contour plots of specific energy absorption (SEA) for BCTs with varying diameters, providing a detailed visualization of how SEA varies with different geometric parameters $A_{i}$ and $A_{j}$. The contour plots indicate that the lowest SEA values are observed at lower $A_{i}$ and higher $A_{j}$ values. This suggests that configurations with smaller initial cell heights ($A_{i}$) and larger lateral expansions ($A_{j}$) are less effective at absorbing energy, likely due to less optimal structural configurations for energy dissipation. As $A_{i}$ increases or $A_{j}$ decreases, SEA progressively increases. This indicates that increasing the initial cell height or reducing the lateral expansion enhances the energy absorption capability of the BCTs. Higher $A_{i}$ values likely contribute to greater structural integrity and improved energy distribution during deformation, while lower $A_{j}$ values reduce the spread of deformation, concentrating energy absorption in a more controlled manner. The intensification of the red regions on the contour plots as the diameter of the BCTs increases highlights an important aspect of their energy absorption performance. The deeper red hues signify higher SEA values, indicating that larger diameter BCTs exhibit greater energy absorption capabilities. This trend can be attributed to the increased material volume and structural capacity of larger diameter tubes, which provide more room for deformation and dissipation of impact forces. Additionally, the red regions shift towards higher values of $A_{i}$ with increasing BCT diameter. This trend indicates that for larger diameter BCTs, higher initial cell heights ($A_{i}$) become more critical in achieving maximum SEA. This shift reflects the necessity for stronger structural configurations in larger diameter tubes to manage and distribute the absorbed energy efficiently.

**Figure 10:**
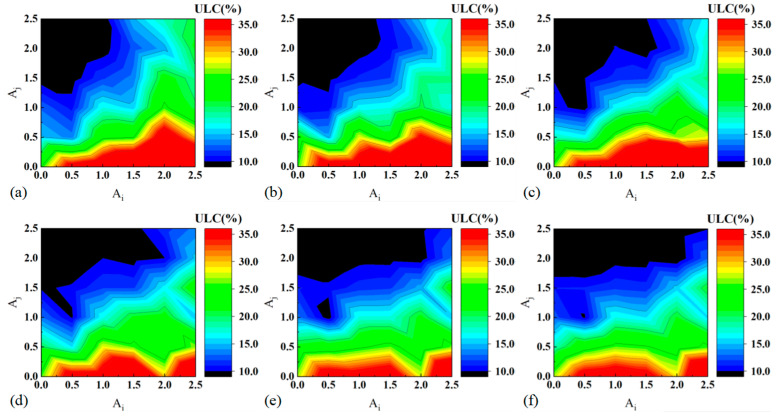
Contour plots of ULC: (**a**) D48, (**b**) D52, (**c**) D56, (**d**) D60, (**e**) D64, and (**f**) D68.

Figure 10 presents the contour plots of ultimate load capacity (ULC) for BCTs with varying diameters, providing detailed insights into how ULC is influenced by the geometric parameters $A_{i}$ and $A_{j}$. The analysis reveals that ULC decreases with increasing $A_{j}$ and decreasing $A_{i}$. This indicates that larger amplitude of axial ripples ($A_{j}$) and smaller amplitude of transverse ripple ($A_{i}$) lead to lower ULC, suggesting reduced structural strength under compressive loads. Notably, $A_{j}$ has a more pronounced effect on ULC compared to $A_{i}$, highlighting its significant role in determining the compressive properties of the BCTs. The contour plots show that the regions of lowest ULC are concentrated within the ranges of $1.5\leq A_{j}\leq 2.5$ and $0\leq A_{i}\leq 1.5$. These ranges indicate the geometric configurations that result in the weakest structural performance under compressive loads. Understanding these ranges is crucial for avoiding designs that may lead to premature failure or inadequate load-bearing capacity. As the diameter of the BCT increases, the area corresponding to the minimum ULC values expands towards higher $A_{i}$ values. This trend suggests that larger diameters require higher $A_{i}$ to maintain structural integrity and avoid significant reductions in ULC. This shift highlights the need to adjust geometric parameters proportionally with the diameter to ensure optimal performance. $A_{j}$ plays a pivotal role in modulating the compressive properties of the BCTs. Its significant impact on reducing ULC compared to $A_{i}$ underscores the importance of lateral expansion in determining the overall structural strength. For optimal deformation stability, $A_{j}$ should be selected at a higher value to minimize buckling effects. This ensures that the BCT can sustain higher compressive loads without experiencing instability or failure.

#### 3.2.3. Comparison between BCTs and CCTs

**Figure 11:**
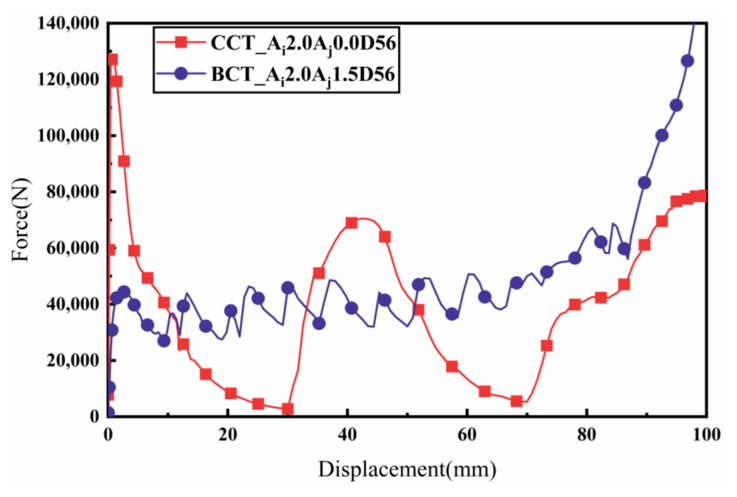
Load–displacement diagrams of the CCT and BCT under axial compressive loading.

**Figure 12:**
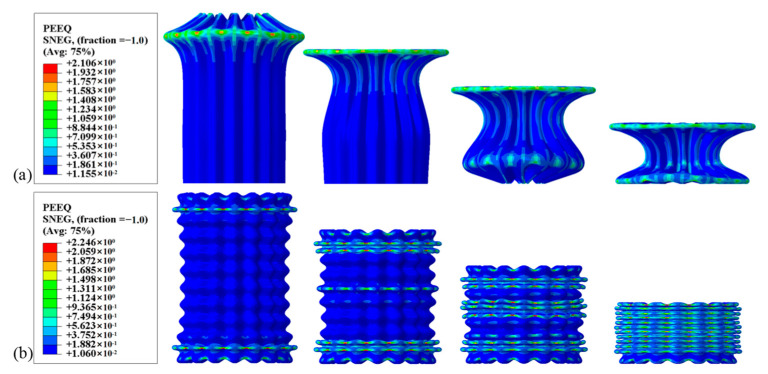
Equivalent plastic strain (PEEQ) contours: (**a**) CCT and (**b**) BCT.

The load–displacement characteristics of BCTs and CCTs with identical geometrical parameters are depicted in Figure 11. The force–displacement curves for CCTs show a rapid increase in force during the initial impact stage, followed by significant fluctuations around the mean load. These fluctuations correspond to the formation of folds, indicating an unstable energy absorption process. In contrast, the intricate cross-section of the BCT enhances its overall axial impact resistance, resulting in a higher initial peak crush force (IPCF). Moreover, BCTs exhibit smaller load fluctuations compared to CCTs, enabling better control over the energy absorption capacity. This controlled deformation results in a more efficient energy absorption process. Further studies indicate that BCTs not only possess a lower ultimate load capacity (ULC), but also achieve a similar or even higher specific energy absorption (SEA) compared to CCTs, making them more efficient in terms of energy absorption. The PEEQ (equivalent plastic strain) contours illustrated in Figure 12 reveal the irreversible plastic deformation induced by the crushing load, which ultimately determines the energy absorption capacity of the tubes [30]. For CCTs, two folds are observed at 28 mm and 92 mm from the moving flat plate, both occurring symmetrically from the horizontal center of the tube and extending towards the ends. This symmetrical folding pattern indicates a localized deformation, which can lead to higher load fluctuations. These findings suggest that BCTs may be more suitable for applications requiring high energy absorption capacity and stable deformation behavior under compressive loads.

In contrast, BCTs exhibit a progressive buckling deformation pattern along the axial corrugation. Pleats initially form at the tube ends, followed by a progressive deformation along the length of the tube. Subsequently, additional pleats emerge from the horizontal center and extend towards the ends. This progressive buckling allows for a more distributed energy absorption process, minimizing the load fluctuations. Notably, the PEEQ regions are concentrated near the crushing interface, indicating effective energy absorption at these points. The irreversible plastic deformation induced by the crushing load is focused in these regions, ultimately determining the energy absorption capacity of the tubes.

The load–displacement response of BCTs exhibits minimal fluctuations during the crushing process, thereby reducing the risk of injury and enhancing crashworthiness. Additionally, the crushing displacement required to form the second fold in BCTs is significantly less than that in CCTs, yet the energy absorption is substantially greater. This indicates that BCTs are capable of absorbing more energy over a shorter deformation distance, enhancing their efficiency. By reducing the required deformation distance, BCTs can potentially enable the design of lighter and more space-efficient crashworthy structures, which is crucial for applications where weight and space are constrained.

Overall, the force–displacement curves of CCTs exhibit significant fluctuations during axial crushing, consistent with Deng’s previous findings [11]. This behavior indicates that CCTs require more energy to develop hinges, which can be attributed to their intricate cross-sectional design that enhances overall structural stiffness. In contrast, BCTs demonstrate a smoother energy absorption pattern due to the axial ripples that dictate the fold locations, allowing the tube to deform in a controlled manner according to the pre-folding, as illustrated in Figure 12.

This figure shows that the specific energy absorption (SEA) of BCTs surpasses that of CCTs by up to 80.23% in the first dataset. This substantial increase in SEA demonstrates that BCTs are more effective at absorbing energy per unit mass compared to CCTs. Simultaneously, the ultimate load capacity (ULC) of BCTs is reduced by 57.63% relative to CCTs. The significant reduction in ULC highlights the BCT’s capability to deform under lower peak loads, which can be advantageous in crash scenarios where controlled deformation is crucial. Here, the SEA of BCTs is shown to be only 11.15% higher than that of CCTs. Despite this relatively modest increase in SEA, the BCT achieves a remarkable ULC reduction of up to 72.96% compared to CCTs. This indicates that BCTs offer a more efficient energy absorption profile, with a substantial reduction in peak loads, which could be beneficial in mitigating impact forces during a crash. These panels further illustrate that BCTs not only maintain a high SEA compared to CCTs of the same size but also exhibit a smoother energy absorption curve throughout the crushing process. The smoother energy absorption is attributed to the BCT’s design, which allows for more controlled and consistent deformation. The axial ripples in BCTs pre-determine the fold locations, enabling the tube to deform in a predictable and stable manner. This results in reduced fluctuations in load and enhanced performance in energy dissipation.

**Figure 13:**
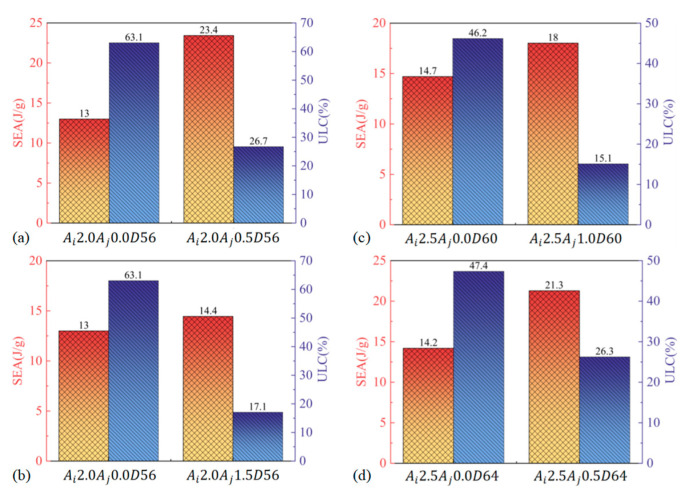
Crashworthiness properties of CCTs and BCTs.

In summary, Figure 13 highlights that BCTs offer superior energy absorption capabilities compared to CCTs, with a higher SEA and lower ULC. The improved performance of BCTs in energy absorption, combined with their ability to maintain smoother load–displacement behavior, demonstrates their potential for enhanced crashworthiness in practical applications.

#### 3.2.4. Comparison between BCTs and LCTs

**Figure 14:**
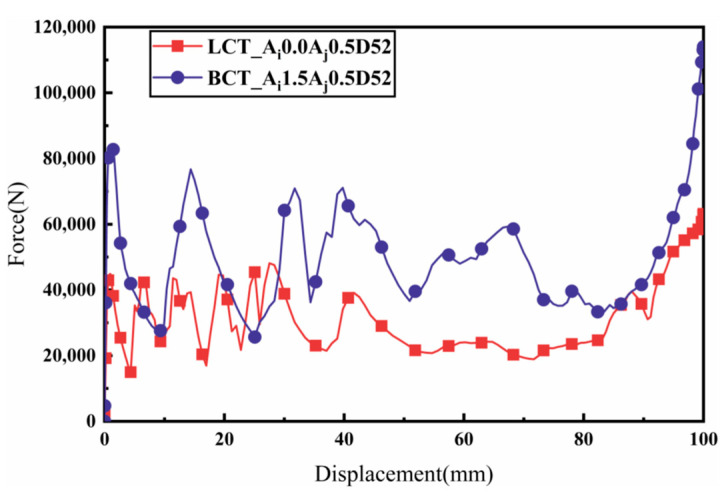
Load–displacement diagrams of the LCT and BCT under axial compressive loading.

The comparative analysis presented in Figure 14 offers profound insights into the load–displacement characteristics of BCTs in contrast to LCTs with identical geometrical parameters. This comparison reveals several key insights into the mechanical performance of BCTs relative to LCTs. Furthermore, the observation that BCTs can withstand augmented loads while maintaining or even reducing their ULC compared to LCTs underscores a notable enhancement in their load-bearing capabilities. Despite having a comparable or lower peak load, BCTs exhibit a unique advantage in their ability to handle higher loads without experiencing significant failure or instability. This is indicative of the BCTs’ efficient load distribution and deformation characteristics. BCTs demonstrate the capacity to withstand higher loads while maintaining or reducing the ULC. This suggests that BCTs offer enhanced load-bearing capabilities and better performance under compressive forces. The improved load resistance of BCTs is likely due to their structural design, which allows for more effective energy absorption and load distribution. The ability of BCTs to maintain similar or lower ULC while withstanding higher loads highlights their potential for applications where both high energy absorption and controlled deformation are required. This makes BCTs suitable for applications such as impact protection and crashworthiness, where efficient energy dissipation and structural resilience are critical. In summary, Figure 14 highlights that BCTs not only match or exceed the performance of LCTs in terms of load-bearing capacity, but also demonstrate the ability to withstand higher loads while maintaining similar or lower ULC values. These findings suggest that BCTs offer significant advantages in terms of mechanical performance and structural efficiency.

**Figure 15:**
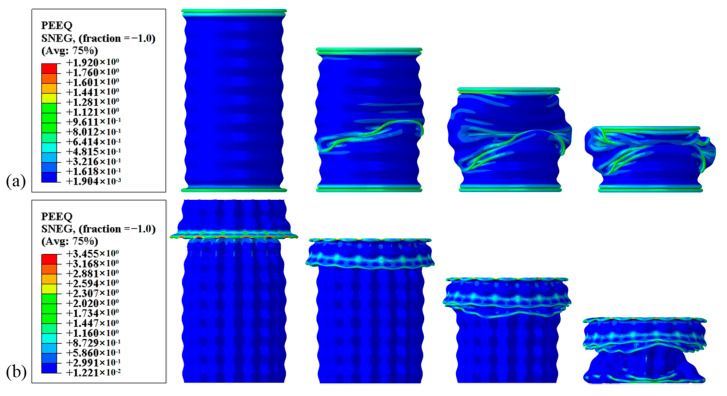
Equivalent plastic strain (PEEQ) contours: (**a**) LCT and (**b**) BCT.

Figure 15 presents a comparative analysis of the PEEQ contours for both the LCT and the BCT, revealing distinct deformation behaviors under compressive loading. The PEEQ contours for the LCT show that the initial two folds form at the extremities of the tube, followed by a significant global buckling deformation at the midpoint. This hybrid deformation pattern indicates that the LCT undergoes localized folding at the ends, which then transitions into a global buckling mode. This results in a less efficient energy absorption process, as the deformation is not uniformly distributed along the length of the tube. In contrast, the BCT initially exhibits folding at a distance of 24.54 mm from the moving plate’s displacement. Following this initial deformation, the successive layers of the unit experience destabilization, with the upper end of each layer moving outward and the lower end moving inward. This local destabilization leads to a global buckling deformation in the lowest four layers of the BCT. This pattern demonstrates a more progressive and controlled deformation, which contributes to more efficient energy absorption. Throughout the crushing process, the load–displacement response of the LCT shows minimal fluctuation but also low energy absorption. This behavior is indicative of the LCT’s less effective energy dissipation, as the hybrid deformation mode results in reduced overall performance in energy absorption. Conversely, the BCT’s load–displacement response exhibits more pronounced fluctuations initially, transitioning to a relatively smooth response. This transition reflects the BCT’s ability to absorb energy more effectively over a larger deformation range, resulting in a significantly higher energy absorption (EA) compared to the LCT. The presence of circumferential corrugation in the BCT enhances its structural stiffness and contributes to a more efficient deformation mode, improving overall crashworthiness. The observed differences in deformation modes between the LCT and BCT highlight the impact of structural design on crashworthiness. The LCT, with its axial corrugation, undergoes a combination of deformation modes that reduce its energy absorption capacity. In contrast, the BCT’s circumferential corrugation improves its structural performance, allowing for more effective energy dissipation and a smoother load–displacement response. These findings underscore the advantages of BCT design in achieving better energy absorption and structural efficiency, consistent with previous studies such as those by Eyvazian et al. [16].

**Figure 16:**
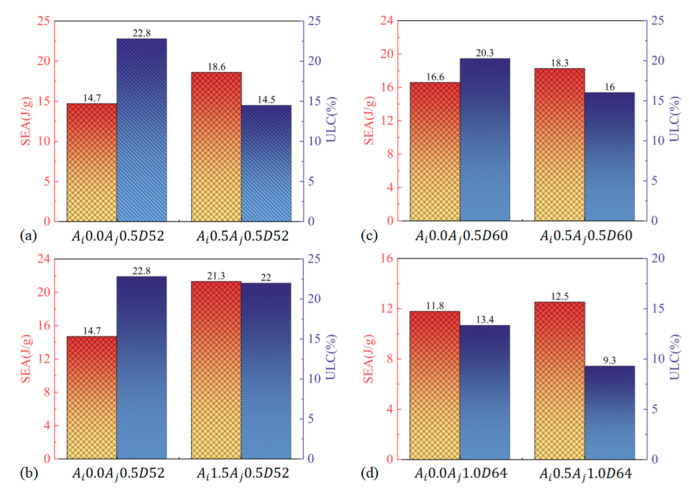
Crashworthiness properties of LCTs and BCTs.

Figure 16 provides a comprehensive comparison of crashworthiness parameters for BCTs and LCTs across four different tube configurations, highlighting the performance differences between these designs. This figure shows that the BCT, with identical geometrical parameters to the LCT, achieves a significant 36.43% reduction in ultimate load capacity (ULC). This significant reduction indicates that the BCT experiences lower peak loads during deformation, which can contribute to reduced risk of catastrophic failure. Additionally, the BCT demonstrates a 36.33% increase in specific energy absorption (SEA), reflecting its superior ability to absorb energy per unit mass. The increase in SEA suggests that the BCT is more effective in dissipating energy during impact, enhancing overall crashworthiness. Here, the BCT exhibits up to 44.9% higher SEA compared to the LCT with the same geometric parameters. This substantial improvement in SEA further underscores the BCT’s effectiveness in energy absorption. The BCT also shows a 3.64% reduction in ULC, reinforcing its capability to handle impact loads more efficiently while maintaining a lower peak load. This combined effect of lower peak loads and heightened energy absorption capabilities positions BCTs as a highly favorable structural solution for applications requiring robust crashworthiness performance, which provides additional confirmation of the BCT’s superior performance in energy absorption. The BCT consistently maintains a lower *ULC* and demonstrates higher SEA compared to the LCT of equivalent size. The results indicate that the BCT offers better overall energy absorption capabilities, which is crucial for applications requiring enhanced impact resistance and controlled deformation. The significant increase in SEA for the BCT compared to the LCT indicates a more efficient energy dissipation mechanism. This improvement is vital for applications where maximizing energy absorption is essential for safety and performance. Furthermore, the reduction in ULC observed in the BCT highlights its advantage in experiencing lower peak loads, which can lead to improved structural performance and reduced risk of failure during impact scenarios. The combination of higher SEA and lower ULC confirms that the BCT outperforms the LCT in terms of crashworthiness. This makes the BCT a more effective choice for applications that require superior energy absorption and load management. In summary, Figure 16 conclusively illustrates that the BCT provides substantial improvements in crashworthiness over the LCT, as evidenced by its higher SEA and lower ULC. These performance metrics underscore the BCT’s exceptional energy absorption capabilities and overall efficiency in mitigating the effects of impact, making it an attractive option for a wide range of demanding applications.

### 3.3. Effect of Wall Thickness

**Figure 17:**
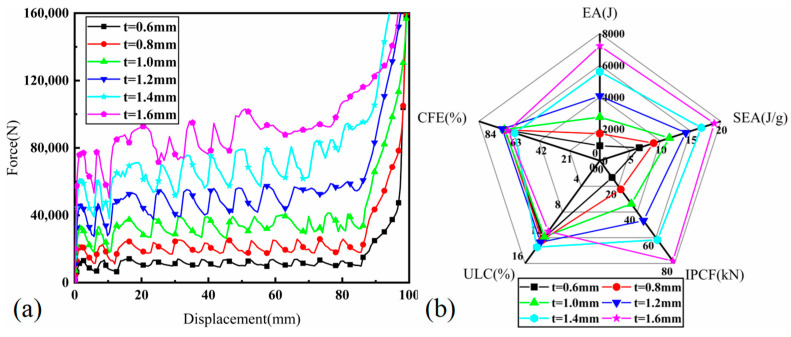
BCT with different thicknesses: (**a**) load—displacement curves; (**b**) radar plot of crashworthiness parameters.

Figure 17a provides a comprehensive illustration of the force–displacement curves for BCTs with varying wall thicknesses ranging from 0.6 to 1.6 mm. As the wall thickness (t) of the BCTs increases, there is a noticeable rise in both the platform force and initial stiffness. This trend indicates that thicker walls contribute to a greater resistance against deformation, resulting in higher peak forces during impact. The increased initial stiffness reflects a more rigid response at the onset of deformation, which is beneficial for applications requiring enhanced structural support. The observed increase in platform force with greater thickness suggests that thicker BCTs can sustain higher loads before yielding or collapsing. This enhancement in load-bearing capacity is critical for applications that demand high impact resistance and durability. Figure 17b presents in a radar plot the crashworthiness parameters of BCTs with different wall thicknesses. ULC remains relatively constant at approximately 12% across different thicknesses. This consistency suggests that the wall thickness has a minimal effect on the maximum load-bearing capacity of the BCTs, implying that other factors may play a more significant role in determining ULC. CFE is around 80% for all thicknesses, indicating a high level of energy absorption efficiency relative to the force applied. This efficiency is maintained across the range of wall thicknesses, demonstrating that the BCT’s design effectively manages impact forces regardless of thickness.

In summary, Figure 17 demonstrates that increasing wall thickness in BCTs enhances their force–displacement response and improves several crashworthiness parameters. While CFE and ULC remain relatively stable, IPCF, EA, and SEA benefit significantly from increased thickness. These findings suggest that optimizing wall thickness is crucial for maximizing the impact performance and energy absorption capabilities of BCTs.

## 4. Optimization

### 4.1. Definition of the Optimization Problem

To assess the impact of parameters on the crashworthiness of the selected tube, the N16M12 configuration was identified as optimal. Variations in the parameter D cause the BCT to transition into the densification stage more rapidly. As an energy absorber, the BCT should maximize the crushing energy per unit mass, making SEA a crucial maximization objective. Additionally, the mechanical response of the energy absorbing structure should exhibit a long and stable stress plateau, making ULC another important metric. A lower ULC indicates a more stable platform force. Thus, the objective is to design a BCT that combines higher SEA with lower ULC. To optimize these crashworthiness metrics, parameters $A_{i}$, $A_{j}$, D, and t are considered variables for optimization. The goal is to achieve high specific energy absorption while maintaining stable energy absorption. Accordingly, a multi-objective optimization framework was established to meet these criteria:

(8) $$\left\{\begin{array}{c} min\left(-SEA,ULC\right)\\ st\;0\;\mathrm{mm}\ll A_{i}\ll 2\;\mathrm{mm}\\ 0\;\mathrm{mm}\ll A_{j}\ll 2\;\mathrm{mm}\\ 50\;\mathrm{mm}\ll D\ll 60\;\mathrm{mm}\\ 0.65\;\mathrm{mm}\ll t\ll 1.35\;\mathrm{mm} \end{array}\right.$$

Here, a simultaneous multi-objective optimization of the CCT and LCT was performed to compare and evaluate the crashworthiness performance of the BCT relative to both the CCT and LCT. The process of multi-objective optimization is outlined as follows:

(9) $$\left\{\begin{array}{c} min\left(-SEA,ULC\right)\\ st\;0\;\mathrm{mm}\ll A_{i}\ll 2\;\mathrm{mm}\\ 50\;\mathrm{mm}\ll D\ll 60\;\mathrm{mm}\\ 0.65\;\mathrm{mm}\ll t\ll 1.35\;\mathrm{mm} \end{array}\right.$$

(10) $$\left\{\begin{array}{c} min\left(-SEA,ULC\right)\\ st\;0\;\mathrm{mm}\ll A_{j}\ll 2\;\mathrm{mm}\\ 50\;\mathrm{mm}\ll D\ll 60\;\mathrm{mm}\\ 0.65\;\mathrm{mm}\ll t\ll 1.35\;\mathrm{mm} \end{array}\right.$$

**Figure 18:**
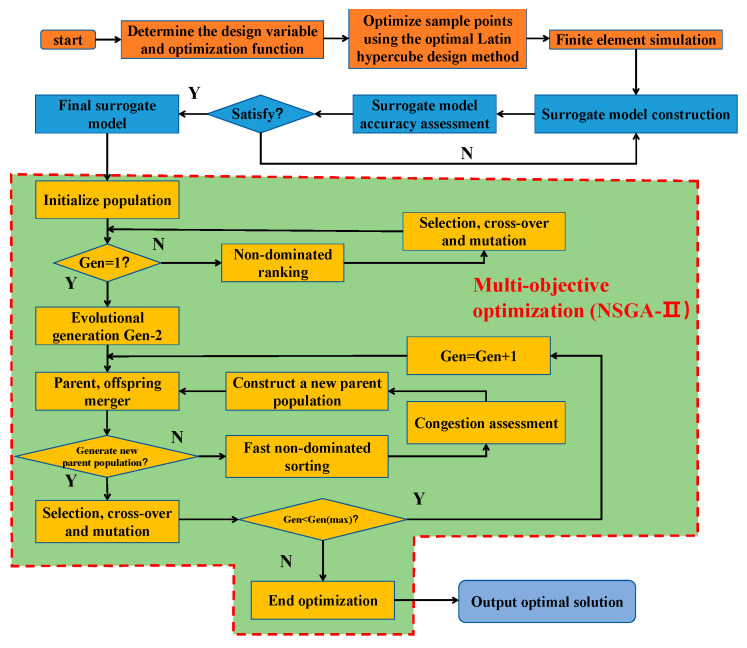
NSGA-II process flow diagram.

The optimization process is detailed in Figure 18. Subsequently, sample points are determined using a design-of-experiments approach and subsequently simulated using ABAQUS/Explicit. The resulting simulation data are utilized to develop a surrogate model through the kriging method. Finally, multi-objective optimization (MOO) is conducted using the NSGA-II algorithm to derive the final Pareto frontier.

### 4.2. Approximate Model

To manage the extensive experimental data, the optimal Latin hypercube design (OLHD) method is employed for determining the sample points, owing to its excellent space-filling and uniform properties. Using this approach, surrogate models for ULC and SEA are developed via the kriging method. Specifically, 51, 21, and 21 sample points are determined for constructing surrogate models for the BCT, CCT, and LCT, respectively, ensuring model accuracy. The crashworthiness parameters corresponding to these sample points are calculated using the finite element method.

Once the surrogate model is constructed, its accuracy is evaluated using a set of 20 sample points. Four evaluation metrics—coefficient of determination ($R^{2}$), relative average absolute error (RAAE), relative maximum absolute error (RMAE), and root mean square (RMSE)—are employed to assess the validity of the constructed model [11]. The specific formulas for these metrics are as follows:

(11) $$R^{2}=1-\frac{\sum_{i=1}^{n}{\left(y_{i}-{\hat{y}}_{i}\right)}^{2}}{\sum_{i=1}^{n}{\left(y_{i}-{\bar{y}}_{i}\right)}^{2}}$$

(12) $$RAAE=\frac{\sum_{i=1}^{n}\left|y_{i}-{\hat{y}}_{i}\right|}{n\times \sqrt{\frac{1}{n-1}\sum_{i=1}^{n}\left(y_{i}-{\hat{y}}_{i}\right)}}$$

(13) $$RMAE=\frac{max\left(\left|y_{1}-{\hat{y}}_{1}\right|,\left|y_{2}-{\hat{y}}_{2}\right|,\ldots ,\left|y_{n}-{\hat{y}}_{n}\right|\right)}{\sqrt{\frac{1}{n-1}\sum_{i=1}^{n}\left(y_{i}-{\hat{y}}_{i}\right)}}$$

(14) $$RMSE=\sqrt{\frac{1}{n}\sum_{i=1}^{n}{\left(y_{i}-{\hat{y}}_{i}\right)}^{2}}$$

Here, $y_{i}$ represents the true values, ${\hat{y}}_{i}$ denotes the predicted values, ${\bar{y}}_{i}$ is the mean value of the sample points, and n is the number of specimens. The model’s accuracy improves as the values of ${1-R}^{2}$, RAAE, RMAE, and RMSE approach 0. Table 3 summarizes the accuracy metrics for the surrogate models of SEA and ULC, demonstrating that the constructed models exhibit high precision.

### 4.3. Optimization Results

**Figure 19:**
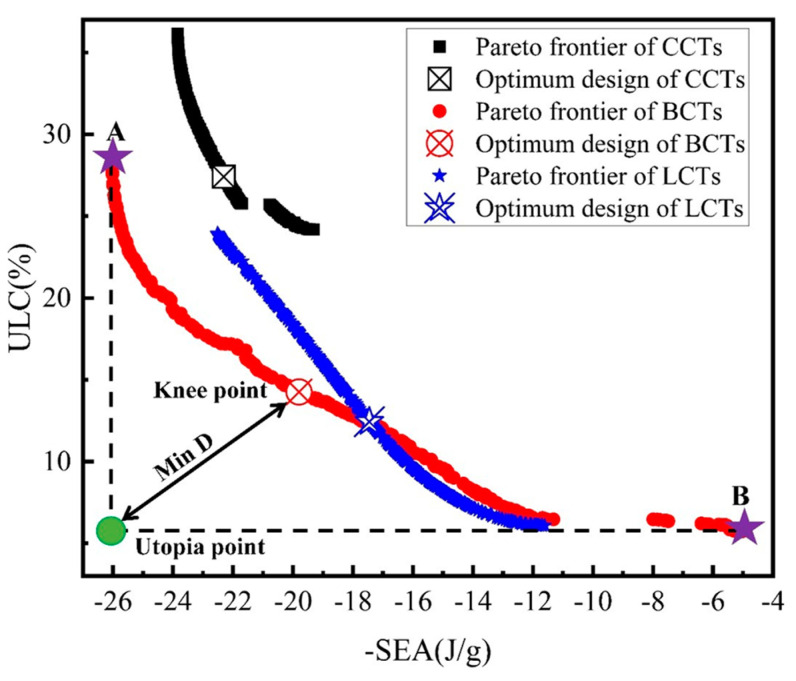
Pareto front of the BCT structure.

The two objectives of the structure are further optimized using the NSGA-II algorithm to derive the Pareto solution set [31]. The optimized Pareto frontier is illustrated in Figure 19. This figure demonstrates that SEA and ULC are conflicting objectives; theoretically, all solutions along the Pareto frontier are optimal and provide valuable guidance for engineers in making decisions based on specific requirements. For instance, point A represents the optimal solution when SEA is prioritized, while point B is ideal for maximizing ULC. The trade-off between SEA and ULC is determined using the minimum distance selection method (TMDSM), which identifies the “knee point” that offers a balanced compromise between the two objectives. The mathematical expression for this compromise point is given by [32]:

(15) $$minD=\sqrt{\frac{1}{n}\sum_{\tau =1}^{n}{\left(\frac{f_{c\tau }-min\left(f_{\tau }\left(x\right)\right)}{max\left(f_{\tau }\left(x\right)\right)-min\left(f_{\tau }\left(x\right)\right)}\right)}^{2}}$$

where $f_{c\tau }$ represents the τth objective value in the Pareto solution for the $c^{th}$ point, MinD is the minimum distance from the “utopia point” to a point on the Pareto frontier, and n is the number of optimization points.

Finally, a comparison between the numerical results and the optimized solutions reveals notable insights, as depicted in Table 4. The maximum discrepancies observed in SEA and ULC between the optimized and numerical results are 2.12% and 3.86%, respectively, indicating a high level of accuracy in the optimized results.

Table 5 presents a comparative analysis of the crashworthiness indices—SEA and ULC—across BCTs, CCTs, and LCTs. When SEA is the primary objective, BCTs demonstrate significant improvements, with increases of 16.72% and 49.14% over optimal CCTs and LCTs designs, respectively. However, these improvements are accompanied by increases in ULC of 3.39% and 126.93%, respectively. Conversely, when minimizing ULC is prioritized, BCTs achieve reductions of 79.09% and 53.94% compared to CCTs and LCTs, respectively, though this results in decreases in SEA of 77.50% and 71.25%, respectively. Notably, the optimal BCT design reduces ULC by 47.99% with only a modest decrease in SEA compared to the optimal CCT design and enhances SEA by 13.40% with a slight increase in ULC compared to the optimal LCT design. These findings highlight the superior crashworthiness of the optimized BCTs through multi-objective optimization.

## 5. Conclusions

To enhance the crashworthiness of thin-walled tubes, this study introduces a novel corrugated structure on both the cross-section and along the wall of the tube, leading to the design of the BCT. The crashworthiness of BCTs was evaluated through axial compression experiments and finite element analysis, yielding the following key conclusions:

1. The BCT is fabricated using a bending method, with the accuracy of the finite element model validated by experimental verification.
2. Discretization analysis assesses the influence of parameters N and M on the crashworthiness of the BCT. The TOPSIS ranking method identifies the optimal values for N and M, which are determined as 12 and 16, respectively.
3. A full-parameter design of experiments investigates the effects of $A_{i}$, $A_{j}$, and D on structural crashworthiness. $A_{i}$ influences the energy absorption capacity, $A_{j}$ enhances the stability of the platform force, and an increase in D improves the likelihood of achieving an optimal energy-absorbing configuration.
4. The SEA of the BCT exceeds that of the CCT by up to 80.23%, while the ULC is reduced by 57.63%. Additionally, the ULC of the BCT is decreased by 72.96% compared to the CCT, and it exhibits a 36.43% lower ULC compared to the LCT, with up to 44.9% higher SEA than the LCT.
5. A simultaneous multi-objective optimization of CCT, LCT, and BCT based on the NSGAII method reveals that the Pareto front of the BCT demonstrates superior crashworthiness advantages compared to both CCT and LCT. The proposed BCT shows significant potential as an energy absorber with promising applications across various fields.

Given its stable reaction load and high SEA value, the BCT holds substantial promise in crashworthiness engineering. In automotive applications, it can be used for the crash box behind the front bumper, and in aerospace, it can be utilized for the bottom plate strut. Further research will focus on analyzing its mechanical properties under high-speed impact and studying its vibration characteristics, ensuring a progressive examination of this work.

## Figures and Tables

**Table 1:** Error analysis of experiment and simulation.

	Mass (g)	MCF (kN)	EA (J)	SEA (J/g)
specimen 1	243.3	30.37	1974.13	8.11
specimen 2	242.8	30.42	1977.18	8.14
FEM	240.99	31.00	2014.98	8.36

**Table 2:** Values of each level of the parameters.

Parameters	Levels	Values
$A_{i}$	6	0, 0.5, 1.0, 1.5, 2.0, 2.5
$A_{j}$	6	0, 0.5, 1.0, 1.5, 2.0, 2.5
D	6	48, 52, 56, 60, 64, 68

**Table 3:** Accuracy evaluation of the surrogate model for BCTs, CCTs, and LCTs.

Columns	Index	$R^{2}$	RAAE	RMAE	RMSE
BCTs	ULC	0.9119	0.0572	0.2391	0.0924
	SEA	0.9370	0.0662	0.1400	0.0793
CCTs	ULC	0.9004	0.1113	0.2176	0.1264
	SEA	0.9357	0.0827	0.1399	0.0917
LCTs	ULC	0.9246	0.0887	0.1632	0.1021
	SEA	0.9704	0.0547	0.1047	0.0622

**Table 4:** Comparison of the optimization value and finite element analysis results.

Item	$A_{i}$	$A_{j}$	D	t	NSGA-II		FEA Results		Accuracies	
					SEA	ULC	SEA	ULC	RE	RE
					(J/g)	(%)	(J/g)	(%)	(%)	(%)
Optimal BCTs	1.55	1.19	51.27	1.35	19.80	14.25	20.22	13.70	2.12	3.86

**Table 5:** Comparison of crashworthiness data for the CCTs, LCTs, and BCTs optimization solution.

Item	Index	$A_{i}$	$A_{j}$	D	t	NSGA-II		RE (Compared to CCTs)		RE (Compared to LCTs)	
						SEA	ULC	SEA	ULC	SEA	ULC
						(J/g)	(%)	(J/g)	(%)	(J/g)	(%)
BCTs	Ideal max SEA	0.93	0.33	54.01	1.35	26.04	28.23	16.72	3.39	49.14	126.93
	Ideal min ULC	0.53	1.86	55.32	0.76	5.02	5.73	−77.50	−79.09	−71.25	−53.94
	Optimal BCTs	1.55	1.19	51.27	1.35	19.80	14.25	−11.25	−47.99	13.40	14.55
CCTs	Optimal CCTs	0.09	0	50.01	1.35	22.31	27.40	-	-	-	-
LCTs	Optimal LCTs	0	0.83	50.00	1.35	17.46	12.44	-	-	-	-

## Data Availability

Data available upon reasonable request.

## Abbreviations

The following abbreviations are used in this manuscript:

LCT	longitudinal corrugated tube
CCT	circumferential corrugated tube
BCT	bi-directional corrugated tube
N	number of units in a single-layer BCT
M	number of layers in the BCT
$A_{i}$	amplitude of transverse ripple
$A_{j}$	amplitude of axial ripples
D	diameter of the neutral plane of the BCT
t	wall thickness of the BCT
H	height of the BCT
r	distance from the XOZ plane
φ	bending angle
R	half of the outer diameter of the BCT
EA	energy absorption
SEA	specific energy absorption
IPCF	initial peak crushing force
MCF	mean crushing force
CFE	crushing force efficiency
ULC	undulation of load carrying capacity
ρ	density
E	Young’s modulus
$\sigma_{y}$	yield stress
ν	Poisson’s ratio
PEEQ	equivalent plastic strain
$F_{max}$	The maximum value of the load in the load displacement curve

## Appendix A

Step one: First create a decision matrix ($D_{x}$) and normalize the data for the $D_{x}$ based on whether the criterion is benefit or cost.

For a problem with n structures and m criteria, $D_{x}$ is constructed as follows:

(A1) $$D_{x}=\left[\begin{array}{cccc} x_{11} & x_{12} & \cdots & x_{1n}\\ x_{21} & x_{22} & \cdots & x_{2n}\\ \vdots & \vdots & \ddots & \vdots \\ x_{m1} & x_{m2} & \cdots & x_{mn} \end{array}\right]$$

where $x_{ij}$ is the performance value of the ith criterion under the jth structure.

The standardized formula is:

(A2) $$s_{ij}=\left\{\begin{array}{c} \frac{x_{ij}-{min}_{i}\left(x_{ij}\right)}{{max}_{i}\left(x_{ij}\right)-{min}_{i}\left(x_{ij}\right)}\left(criteria\;is\;benefit\right)\\ \frac{{max}_{i}\left(x_{ij}\right)-x_{ij}}{{max}_{i}\left(x_{ij}\right)-{min}_{i}\left(x_{ij}\right)}\left(criteria\;is\;cost\right) \end{array}\right.$$

where ${max}_{i}\left(x_{ij}\right)$ and ${min}_{i}\left(x_{ij}\right)$ stand for the maximum and minimum values of the samples under the ith criterion, respectively.

According to the above equation, the standardized matrix $S={\left(s_{ij}\right)}_{mn}$ can be obtained.

Step two: Normalize each row of the normalized matrix and calculate the entropy value and weights.

The normalized formula is:

(A3) $$n_{ij}=\frac{s_{ij}}{\sum_{i=1}^{n}s_{ij}}$$

The entropy formula for the ith standard is:

(A4) $$e_{i}=-\frac{1}{\ln n}\sum_{i=1}^{n}n_{ij}\ln n_{ij}$$

The weights under each criterion are calculated separately after calculating the entropy value:

(A5) $$w_{i}=\frac{1-e_{i}}{\sum_{i=1}^{m}\left(1-e_{i}\right)}$$

Step three: Construct a weighted matrix $W={\left(w_{ij}^{*}\right)}_{mn}$ and obtain two ideal solution points.

The weighted matrix is calculated as:

(A6) $$w_{ij}^{*}=w_{i}\times s_{ij}$$

The ideal solution points are the maximum and minimum values of under each criterion, respectively:

(A7) $$W^{+*}=\left\{w_{1}^{+*},w_{2}^{+*},\cdots ,w_{m}^{+*}\right\}=\left\{w_{1},w_{2},\cdots ,w_{m}\right\}$$

(A8) $$W^{-*}=\left\{w_{1}^{-*},w_{2}^{-*},\cdots ,w_{m}^{-*}\right\}=\left\{0,0,\cdots ,0\right\}$$

Step four: Euclidean distance between each scenario and the ideal scenario with the relative proximity of the alternatives is calculated.

Euclidean distance formulas are, respectively:

(A9) $$D_{j}^{+}=\sqrt{\sum_{i=1}^{m}{\left(w_{i}^{+*}-w_{ij}^{*}\right)}^{2}}=\sqrt{\sum_{i=1}^{m}{\left(w_{i}-w_{ij}^{*}\right)}^{2}}$$

(A10) $$D_{j}^{-}=\sqrt{\sum_{i=1}^{m}{\left(w_{i}^{-*}-w_{ij}^{*}\right)}^{2}}=\sqrt{\sum_{i=1}^{m}{\left(w_{ij}^{*}\right)}^{2}}$$

The formula for relative proximity is:

(A11) $$Q_{j}=\frac{D_{j}^{-}}{D_{j}^{+}+D_{j}^{-}}=\frac{\sqrt{\sum_{i=1}^{m}{\left(w_{ij}^{*}\right)}^{2}}}{\sqrt{\sum_{i=1}^{m}{\left(w_{i}-w_{ij}^{*}\right)}^{2}}+\sqrt{\sum_{i=1}^{m}{\left(w_{ij}^{*}\right)}^{2}}}$$

Finally, $Q_{j}$ is sorted from highest to lowest.

## Appendix B

**Table A1:** Ranking using the entropy TOPSIS method.

Tubes	$Q_{j}$	Ranking	Tubes	$Q_{j}$	Ranking
N4M6	0.0076	19	N12M10	0.2954	11
N4M8	0.2060	16	N12M12	0.3533	6
N4M10	0.2816	13	N16M6	0.3486	7
N4M12	0.2901	12	N16M8	0.3057	10
N8M6	0.1760	18	N16M10	0.5116	5
N8M8	0.2518	15	N16M12	0.6022	4
N8M10	0.3227	9	N20M8	0.6435	3
N8M12	0.3270	8	N20M10	0.8101	1
N12M6	0.1766	17	N20M12	0.7906	2
N12M8	0.2621	14			
